# Synthesis and Application of H-ZSM-5 Zeolites with Different Levels of Acidity as Synergistic Agents in Flame Retardant Polymeric Materials

**DOI:** 10.3390/polym11122110

**Published:** 2019-12-16

**Authors:** Felipe Reis Bernardes, Michelle Jakeline Cunha Rezende, Victor de Oliveira Rodrigues, Regina Sandra Veiga Nascimento, Simone Pereira da Silva Ribeiro

**Affiliations:** Universidade Federal do Rio de Janeiro, Instituto de Química, Cidade Universitária, CT, Bloco A, Rio de Janeiro 21941-909, Brazil; felipereis314@gmail.com (F.R.B.); mjcrezende@gmail.com (M.J.C.R.); vicerodrigues@gmail.com (V.d.O.R.); rsandra@iq.ufrj.br (R.S.V.N.)

**Keywords:** H-ZSM-5 zeolite, acidity, flame retardant, intumescent formulations, polypropylene, synergistic agents

## Abstract

Several studies show a synergistic effect between intumescent formulations and aluminosilicates, such as zeolites and clays, but little is known about the effect of acidity of these additives on the synergistic action. In this work, H-ZSM-5 zeolite was submitted to desilication treatments for 30 min and for 2 h, and silicalite-1 was synthesized. The objective was to obtain samples of equivalent crystalline structure, but with different amounts of acid sites, in order to evaluate the effect of acid concentration of H-ZSM-5 zeolites on the synergistic action with an intumescent formulation composed by ammonium polyphosphate and pentaerythritol in polypropylene. H-ZSM-5 zeolites and silicalite were characterized by X-ray diffraction, nitrogen adsorption analysis and temperature-programmed desorption of ammonia. The desilication produced H-ZSM-5 zeolites with similar volumes of mesopores in both treatments, but the zeolite resulting from 2 h of desilication presented a higher concentration of acid sites than the zeolite from 30 min. The flame-retardant properties were evaluated by UL-94 classification, limiting oxygen index, glow-wire, thermogravimetric analysis and heating microscopy. The results showed that increasing the concentration and accessibility of the acid sites of H-ZSM-5 zeolites the flame-retardant properties of the studied composites improved. It is suggested that the increase of acid site concentration positively influences the catalysis of the reaction between ammonium polyphosphate and pentaerythritol, favoring the production of the precursors of the intumescent layer.

## 1. Introduction

Polypropylene (PP) is indispensable for society, finding wide use every day. However, it has high flammability due to its aliphatic structure, with high content of carbon and hydrogen which, in combustion, quickly diffuses flames, drips, and releases a lot of heat [1,2,3,4,5,6]. As a result, the development of flame-retardant polymeric materials has been growing and gaining prominence.

Among the flame-retardant additives, intumescent formulations are becoming increasingly important. They are halogen free, have low toxicity, and have been widely applied in PP [7]. The intumescent formulation is usually composed of three active components: an acid source such as ammonium polyphosphate (APP), which generates acidic species during its thermal degradation at temperatures below 280 °C, a carbonaceous agent, which is generally a polyhydroxylated compound such as pentaerythritol (PER), and a blowing agent, which throughout its thermal degradation releases non-flammable gases, such as ammonia, causing swelling of the system, and forming a superficial and protective intumescent layer called *char* [8,9,10]. This phosphocarbonaceous material is formed by macromolecular and crystalline cells of polyaromatic rings linked by fragments of polymeric chain and phosphate groups [11,12]. The *char* produces a protective barrier that prevents the diffusion of heat, oxygen and fuel, leading to self-extinguishing of the flame [13,14]. However, intumescent formulation alone does not lead to the commercially desired flame-retardant properties. At temperatures above 400 °C, due to its low thermal stability, cracking begins to occur in the intumescent layer, exposing the composite to flame again [15,16,17]. 

Several studies have focused on the development of synergistic agents that increase the effectiveness of intumescent formulations, some examples include: organometallic compounds [18], montmorillonite [19,20,21], and zeolites [22,23]. Studies have shown that montmorillonite, faujasite, mordenite and 4A zeolites act as synergistic agent in intumescent formulations, catalyzing the reactions of carbonaceous layer formation and improving the mechanical properties of the protective layer, increasing its thermal stability [12,21]. According to the authors, the addition of zeolite maintains the amorphous chains between polyaromatic structures at temperatures above 550 °C, preventing the overgrowth of the crystalline domain, and thus promoting good mechanical properties to the *char* [12,22,23,24,25,26]. Despite the large number of publications on the use of zeolites as synergistic agents, little has been discussed regarding the effect of their acidity on the synergistic action with an intumescent formulation. Recently, our research group showed that faujasite zeolites with higher concentrations of moderate-strength acid sites could catalyze the reaction between APP and PER with higher efficiency, producing phosphate esters (precursors of the *char*) and improving flame-retardant properties [27]. On the other hand, an excessive increase in the strength of acid sites shows the opposite effect, as the zeolite may be prematurely deactivated during the initial stages of the formation of *char*. It is important to highlight that for the production of *char*, it is necessary that the phosphate esters form unsaturated and cyclic structures as they thermally degrade [13,14,28]. 

The catalytic activity of zeolites depends on the number of acid sites and their accessibility [29]. More efficient use of zeolite-based catalysts could occur if their diffusional limitations in the microporous network could be minimized by mesopore formation. Techniques such as desilication have gained prominence because they allow the increase of mesopores without reducing too much the microporosity. Desilication can enhance the acidity of H-ZSM-5 zeolites, for example, by decreasing Si/Al ratio, increasing the amount of Brønsted acid sites, which are also more available due to the higher volume of mesopores [30]. 

H-ZSM-5 zeolite, particularly, has been widely employed as heterogeneous catalyst due to a combination of properties such as: high specific area, well-defined porosity, high thermal stability, intrinsic acidity and the ability to confine metallic active species [29,31]. Despite its wide application in catalysis, its use as a synergistic agent in intumescent formulations has still been poorly studied [32]. This work reports the effect of the concentration and accessibility of acid sites of H-ZSM-5 zeolites on synergistic action with an intumescent formulation. Desilication of H-ZSM-5 zeolite was carried out under two different conditions, in order to increase the acidity without excessive loss of crystalline structure. Silicalite with analogous crystalline structure [29], but without aluminum in its composition, was also synthesized for the study. Polypropylene composites with intumescent formulation composed by APP, PER and the respective zeolites were evaluated in relation to their flammability and thermal stability using UL-94 classification, limiting oxygen index, glow-wire test, thermogravimetric analysis and heating microscopy technique. H-ZSM-5 zeolites and silicalite-1 were characterized concerning their crystalline structure, textural properties and acidity.

## 2. Materials and Methods

### 2.1. Process of Desilication

NH_4_^+^-ZSM-5 zeolite (Zeolyst CBV 5524G, surface area 425 m^2^ g^−1^, SAR = 50) was calcined for 4 h at 400 °C to form H-ZSM-5 zeolite, called zeo1. Zeo1 was then used as starting material in the process of desilication according to the method previously described by Groen et al. [29]. 2 g of zeo1 were treated with 100 mL of 0.3 M NaOH solution for 30 min. Next, three consecutive ion exchanges were carried out with 100 mL of 1 M NH_4_Cl solution at 60 °C for 3 h. The sample was calcined at 500 °C under dry air flow at 200 mL min^−1^ for 4 h. The final product was called zeo2. The procedure was performed again, but treating H-ZSM-5 zeolite (zeo1) with NaOH solution for 2 h. Ion exchange and calcination steps were done as previously described. The final product was called zeo3. 

### 2.2. Synthesis of Silicalite-1

To estimate the ZSM-5 zeolite with the lowest possible acidity, a silicalite-1 with the same type of structure was synthesized. The synthesis was performed as described by Watanabe et al. [33]. 12.917 g of tetrapropylammonium hydroxide (TPAOH) and 0.015 g of NaOH were added in 9.716 g of water. The solution was kept under constant stirring for 10 min and then 7.352 g of tetraethyl orthosilicate (TEOS) was added dropwise. The SiO_2_/TPAOH/NaOH/H_2_O/EtOH molar ratio in the solution was 1:0.36:0.0104:31.53:4. This solution was stirred for 4 h, and then it was transferred to a closed 40 mL Teflon flask and kept in an oven at 170 °C for 7 days. The obtained solid was calcined at 500 °C for 5 h. The final product was called sil.

### 2.3. Characterization of H-ZSM-5 Zeolites and Silicalite-1

#### 2.3.1. X-Ray Diffraction Analysis

The crystallinity was evaluated by X-ray diffraction (XRD, Rigaku, Tokyo, Japan). The analysis was performed on a Rigaku Miniflex with Cu Kα radiation (λ = 0.15418 nm) at 40 kV and 15 mA. A 2θ range from 5° to 80° was scanned at 0.02° s^−1^. 

#### 2.3.2. Nitrogen Adsorption Analysis

BET-specific surface area was measured by physisorption of nitrogen at 196 °C, employing a Micromeritics A.S.A.P. 2010, Narcron, GA, USA [34]. The average pore diameter was calculated using BJH method and the volume of mesopores and micropores was calculated by t-plot method [35]. Prior to each measurement, all samples were degassed at 300 °C under vacuum until a minimum degassing rate of 2 µm Hg min^−1^.

#### 2.3.3. Temperature-Programmed Desorption of Ammonia (NH_3_-TPD)

The acidity of the samples was determined by desorption of NH_3_ at programmed temperature in a Zeton-Altamira AMI-90 instrument, Pittsburgh, PA, USA. 0.1 g of sample was pretreated at 500 °C under helium flow rate of 30 mL min^−1^, and then cooled to 35 °C for the start of the analysis. Adsorption was done with pulses of ammonia at 175 °C. Desorption was performed up to 500 °C with a heating rate of 10 °C min^−1^ under helium flow. Finally, the sample was kept at 500 °C for 120 min.

### 2.4. Processing of the Composites

The polymeric matrix used in this work was polypropylene (PP), supplied by Braskem (EP 448R code). The intumescent formulation was composed by ammonium polyphosphate (APP), purchased from Clariant, Rio de Janeiro, Brazil (Exolit AP422 code), and pentaerythritol (PER) provided by Sigma Aldrich (Rio de Janeiro, Brazil). The polymer composites contained 30% (*w*/*w*) of formulation with APP/PER mass ratio of 3:1 [32]. The H-ZSM-5 zeolites (zeo1, zeo2 and zeo3) and silicalite (sil) composed 3% (*w*/*w*) of the total mass of the polymeric mixture.

The composites were processed in a Haake Rheomex OS PTW16 double screw extruder (Waltham, MA, USA) with 300 rpm screw rotation. The temperatures of the heating zones were: T_1_ = 90 °C; T_2_ = 135 °C; T_3_ = 170 °C; T_4_ = 180 °C; T_5_ = 190 °C; and T_6_ = 190 °C. Then, the composites were pelleted and pressed into 100 mm × 100 mm × 3 mm size plates. The pressing was performed on a Carver press at 220 °C for 4 min under 6000 lbf, followed by additional 6 min under 12,000 lbf. Finally, a cold press was performed at 15,000 lbf for 5 min.

### 2.5. Evaluation of the Flame-Retardant Properties

#### 2.5.1. UL-94 Classification

The ease of flammability of the composites was evaluated by UL-94 method (ANSI/ASTM D 635-77). In this test, a strip of the material is held vertically in air and ignited from the bottom using a standard technique. The material was classified according to its burning characteristics and time required for extinguishing after flame removal. To be classified as V0, the material must self-extinguish quickly and cannot produce burning drips.

#### 2.5.2. Limiting Oxygen Index (LOI)

A Fire Testing Technology instrument (East Grinstead, UK) was employed with a 100 mm × 7 mm × 3 mm specimen, following ISO 4589-2 standard method. Measurements are accurate to ±1 LOI unit. The test points out the minimum amount of oxygen needed for the sample to ignite and sustain burning in the vertical position. The higher the LOI value, the more flame-resistant the material is.

#### 2.5.3. Glow-Wire Test

The glow-wire technique evaluates the flammability of polymeric materials used in electrical components [36]. Thermal stress could be generated in electro technical systems due to inadequate installations or overload in its components. A CEAST (Pianezza, Italy) 6447A instrument provided the contact of an incandescent tip with a 70 mm × 7 mm × 7 mm specimen, for 30 s, heated by an electrical resistance under the conditions specified in IEC 60695-2-10, IEC 60695-2-12 and IEC 60695-2-13 standards. The test temperature ranges from 550 to 960 °C. Two tests were performed: GWFI (glow-wire flammability index), which corresponds to the highest testing temperature at which the material does not exhibit flame or incandescence for more than 30 s after incandescent tip removal and also does not ignite, through material dripping, the paper positioned below the specimen; and GWIT (glow-wire ignition temperature), which is the temperature 25 °C above the highest temperature tested at which ignition of the material does not occur in three consecutive measurements.

#### 2.5.4. Thermogravimetric Analysis

Thermal analysis can contribute to the evaluation of flammability of polymeric materials although it is not a specific technique for this purpose [37,38,39,40]. After processing, the samples were pulverized in a cryogenic mill and submitted to thermogravimetric analysis (TGA). The thermal stability of the composites was determined using a TA Instruments (New Castle, DE, USA) SDT-Q600. The analysis was performed under the following operation condition: 15 mg sample were submitted to a temperature ramp from 35 to 850 °C in a platinum pan, with 40 °C min^−1^ heating rate and 50 cm^3^ min^−1^ synthetic air flow. 

#### 2.5.5. Heating Microscopy Analysis

The behavior of the material under heating and the preservation of the intumescent layer structure were observed by the technique [41]. The analyses were carried out in a Leitz (Oberkochen, Germany) heating microscope (model 1A) and the images were captured by a Samsung SDC 414 ND camera. The material (a cube with 3 mm sides) was heated from 30 to 900 °C, using a heating rate of 40 °C min^−1^.

## 3. Results and Discussion

### 3.1. Characterization of H-ZSM-5 Zeolites and Silicalite-1

The H-ZSM-5 zeolite used as a starting material (zeo1), the two H-ZSM-5 zeolites resulting from the desilication treatment (zeo2 and zeo3), and the synthesized silicalite-1 (sil) were characterized with respect to their crystalline structure, textural properties, and amount of acid sites. The characterization data are presented and discussed below.

#### 3.1.1. X-Ray Diffraction Analysis

To evaluate the integrity of the zeolites and silicalite structures, XRD analyses were performed. Crystallinity values were estimated from peak areas between 22° and 25°, assuming zeo1 as a 100% crystalline standard, and the crystallinity for other samples proportionally calculated.

**Figure 1:**
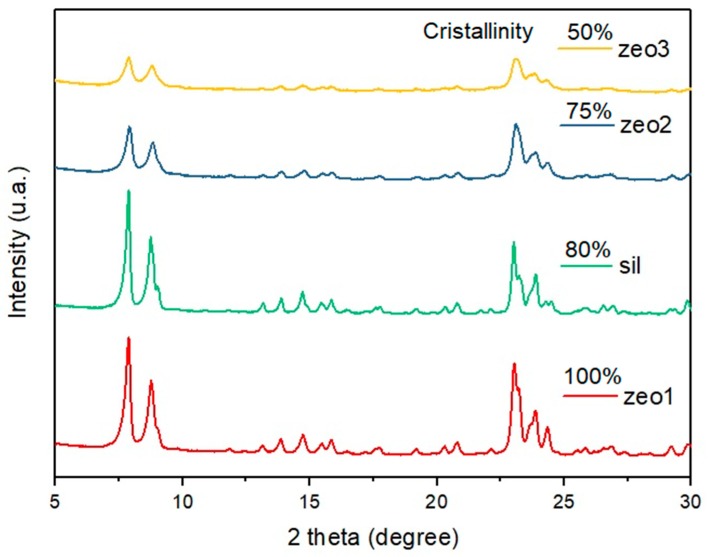
XDR patterns of the samples of H-ZSM-5 zeolites and silicalite.

As expected, the crystallinity decreased due to desilication treatment [29], but the ZSM-5 structure was kept as seen in Figure 1. In sample zeo3, the increase in treatment time led to a lower final crystallinity, but this was still above 50% of the initial (zeo1) value.

The XRD analysis is also presented for the silicalite sample in order to verify if the ZSM-5 structure was obtained. The overall profile and peak symmetries were equal to the standard used in this work (zeo1), indicating that these samples share the same structure. It is important to note that silicalite has higher crystallinity than zeo2 and zeo3, and therefore possible changes in the synergistic properties with the intumescent formulation should be intrinsically related to acid site concentration and to the micro/mesopore volume ratios.

#### 3.1.2. Nitrogen Adsorption Analysis

**Figure 2:**
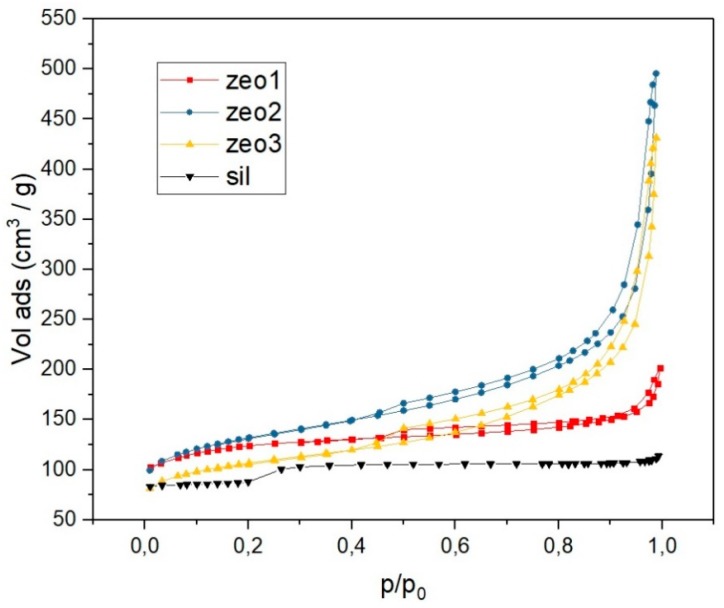
Physisorption isotherms for H-ZSM-5 zeolites and silicalite.

Figure 2 shows nitrogen physisorption isotherms at 196 °C for the H-ZSM-5 zeolites and silicalite used in this work. The zeo1 sample isotherm shows a plateau in a broad range of pressures, which is characteristic of a type I isotherm [42]. Analyzing isotherms from samples zeo2 and zeo3, it is possible to observe that the alkaline treatment changed the isotherm classification, similar in both zeolites, a mixture of isotherm types I and IV, with a plateau at lower and intermediate relative pressures, and a steep ramp at higher relative pressures. Type IV isotherms are typical in mesoporous materials, with a more prominent hysteresis loop. Mesopore development can be verified in Table 1, where the mesopore volume increased from 0.14 cm^3^ g^−1^ in zeo1 (which probably consists of the macropore contribution) to 0.40 and 0.46 cm^3^ g^−1^ in samples zeo3 and zeo2, respectively. Analyzing the specific surface areas (SSA) shown in Table 1, it is possible to observe the generation of mesopores without significant changes in the surface area. The same can be concluded with respect to the zeolites′ micropore volumes. Concerning the silicalite sample, a highly crystalline material with slightly lower specific surface area than its aluminosilicate counterparts was synthesized. Silicalite physisorption isotherm is type I with an SSA of 294 m^2^ g^−1^ and an almost imperceptible hysteresis loop, a consequence of the long crystallization period used [33].

#### 3.1.3. Temperature-Programmed Desorption of Ammonia (NH_3_-TPD)

**Figure 3:**
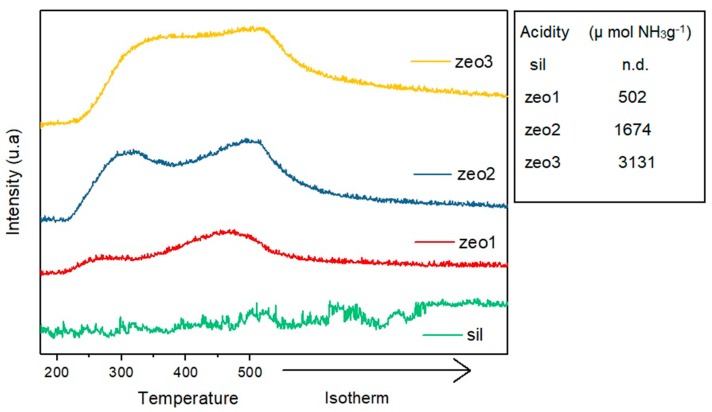
Temperature-programmed desorption of ammonia (NH_3_-TPD) profiles and total acidity of H-ZSM-5 zeolites and silicalite.

Ammonia desorption curves and total acidity for all samples are shown in Figure 3. With the exception of silicalite, two ammonia desorption peaks were observed in all samples, indicating the presence of acid sites with different strengths. Ammonia desorbed at lower temperatures is more loosely attached to the samples surfaces and correlates with weaker acid sites. The opposite can be said to ammonia desorbed at higher temperatures. The silicalite sample presented no peaks, only a stable base line evidencing the absence of acid sites, which was already expected from the absence of aluminum [43].

Ammonia desorption profiles and total acid site concentrations show that the desilication treatment leads to an increase in zeolites acidities, from 502 μmol g^−1^ in the untreated H-ZSM-5 zeolite (zeo1) to 1674 and 3131 μmol g^−1^ in the alkaline treated samples, zeo2 and zeo3, respectively.

It can also be noted that the desilication treatment did not change the desorption profile and therefore the acid strength distribution in sites, but increased the acid sites concentration, as shown by the overall peak areas. In other words, although increasing the alkaline treatment from 30 min (zeo2) to 2 h (zeo3) did not further increase the mesopore volume, it led to an increase in the acid site concentration.

### 3.2. Evaluation of Flame-Retardant Properties

The flame-retardant properties were evaluated by UL-94 classification, limiting oxygen index, glow-wire, thermogravimetric analysis and heating microscopy. The results are discussed below.

#### 3.2.1. UL-94 Classification

UL-94 is a test where V0 is the desired classification, indicating that the specimen self-extinguishes the flame more effectively and that there is no dripping of the burning material. Table 2 presents the results obtained for polypropylene composites with and without the intumescent formulation.

It can be observed that the addition of H-ZSM-5 zeolite (zeo1), modified zeolites (zeo2 and zeo3) and silicalite (sil) to the polypropylene in the absence of intumescent formulation did not lead to any classification of the composite, as it does not self-extinguish the flame, burning up to the holder with dripping of material in flame. The addition of intumescent formulation to polypropylene, in contrast, raised the result in maximum classification (V0). This effect has previously been reported by other authors [44,45]. The addition of the different zeolites and silicalite kept the maximum classification. Thus, the presence of the inorganic material, regardless of its acid characteristics, does not interfere in the classification obtained by the sample PP + APP/PER. 

#### 3.2.2. Limiting Oxygen Index (LOI)

The limiting oxygen index (LOI) of the samples is shown in Table 3. The addition of zeolites with different concentrations of acid sites did not change the result of LOI compared to pure PP, which remained at 17%. Silicalite, which had the same crystalline structure of H-ZSM-5 zeolite, but had a minimum acidity value because it did not have aluminum in its structure, also did not increase the value of LOI. Similar results were observed for polypropylene with sodium 4A and faujasite zeolites, and also with other polymer matrices [21,27]. Thus, the result indicates that the use of zeolites alone, regardless of their crystalline structure and concentration of acid sites, does not improve flame-retardant properties. 

On the other hand, the addition of intumescent formulation composed by APP-PER to the polymer increased LOI from 17% to 31%, as previously observed by other authors [44]. The addition of H-ZSM-5 zeolite (zeo1), which had acid sites, to the intumescent formulation raised the LOI to 33% indicating a synergistic effect. The addition of zeo2 to the intumescent formulation did not promote significant LOI alteration, considering the accuracy of the technique. Zeo2 had a concentration of acid sites three times higher when compared to zeo1 and also a greater accessibility of these sites, observed by the ratio between the volume of mesopores and micropores. In contrast, when zeo3 was added, a significant increase in LOI was observed, to 35%. Zeo3 had a ratio of mesopore and micropore volumes similar to that of zeo2, which indicated that the accessibility to acid sites was retained. However, the concentration of acid sites was almost twice as high as that of zeo2. The LOI value obtained for the composite containing silicalite, which did not have acid sites in its structure, indicated the absence of synergistic action with APP-PER. These results showed that the presence of acid sites in H-ZSM-5 zeolites is fundamental for the occurrence of synergistic action with the intumescent formulation. Furthermore, it is assumed that increasing accessibility and concentration of acid sites to certain values may further elevate the synergistic action between H-ZSM-5 zeolites and the intumescent formulation.

In a previous study by our research group, it was observed that increasing the strength of acid sites of faujasites in PP + APP/PER composites decreased the synergistic action measured by LOI, probably due to a premature deactivation in the early stages of the formation of *char* [27]. In the present study, zeolites have acid sites of similar strength, differing only in their concentration and accessibility. Thus, it can be supposed that increasing the concentration of acid sites and their accessibility may enhance the catalysis of the esterification reaction between APP and PER, leading to more effective formation of the intumescent layer precursors, having a positive impact on the flame-retardant properties.

Considering that the structure of zeolites can collapse at high temperatures and in contact with very acid solutions, it may be suggested that during the formation of the intumescent layer, the destruction of the zeolite crystalline structure occurs due to the production of phosphoric acid, from the thermal decomposition of ammonium polyphosphate, which occurs around 280 °C. Bourbigot et al. verified the formation of aluminosilicophosphoric species above 280 °C by ^27^Al NMR, which would promote greater thermal stability for the intumescent layer [22]. This type of structure has also been observed in composites of PP + APP/PER + montmorillonite heated at around 280 °C [20]. Therefore, taking into account a possible collapse of the crystalline structure at 280 °C, it is suggested that zeolites should act at first catalyzing the esterification reaction, where the concentration and accessibility of acid sites are determinant, and later in the formation of aluminophosphoric species, increasing the thermal stability of the protective layer. 

#### 3.2.3. Glow-Wire Test

The GWFI (Glow-wire flammability index) and GWIT (Glow-wire ignition temperature) values of the composites containing the intumescent formulation and the zeolites under study are shown in Table 4. This test is typical for polymeric materials used in the electrical sector, but despite its importance, there are few published studies using this technique. For polymeric materials in contact or near electrical conductors, where the current is greater than 0.2 A, the maximum GWFI is 850 °C and the minimum is 775 °C [46]. Thus, it can be seen that pure PP, with a GWIT value of 700 °C, did not reach the minimum value recommended. 

On the other hand, the addition of the intumescent formulation enhanced the GWFI to 850 °C and the addition of zeolites, regardless of acid site concentration and accessibility, increased the value of the standard to a maximum of 960 °C. Furthermore, the addition of the intumescent formulation also increased the GWIT of the polymer. In this parameter, it was observed that the expressive increase of the accessibility and concentration of acid sites, verified in the samples zeo2 and zeo3, augmented the GWIT, reaching the value of 875 °C. This result was also evidence that the significant increase in accessibility and concentration of acid sites may favor the production of the precursors of the intumescent layer by reaction between APP and PER, leading to the formation of the intumescent layer in a shorter time, and therefore increasing the temperature of ignition (GWIT). In contrast, the composite containing silicalite exhibited the same GWIT of the one containing zeo1, which is still 50 °C higher than the PP + APP/PER composite. This result indicates that PP + APP/PER composites with H-ZSM-5 zeolites that have lower concentration of acid sites and lower accessibility to these sites have the same GWIT value of the composite containing silicalite, which has no acid sites, but the same crystalline structure as H-ZSM-5. The accessibility of acid sites may be a major factor for synergistic action, since an increase in GWIT was observed when zeo2 or zeo3 were added to the formulation, i.e., when the volume of mesopores increased. In addition to that, the addition of zeo1, which had less accessible acid sites, led to the same ignition temperature as the composite containing silicalite, which had no acid sites.

#### 3.2.4. Thermogravimetric Analysis

**Figure 4:**
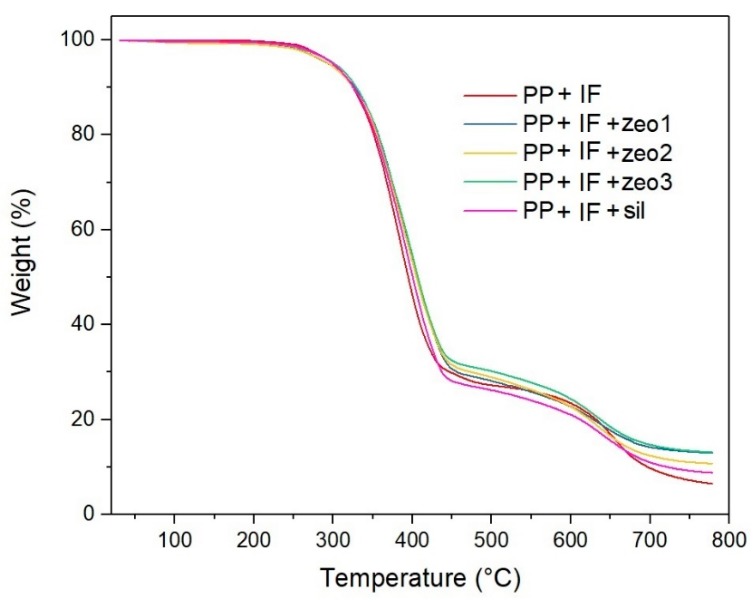
TGA curves of composites of polypropylene with intumescent layer.

TGA has been used in the evaluation of various composites with flame-retardant properties [47,48,49]. Figure 4 shows the TGA curves of composites with intumescent formulation (IF). It can be observed that the addition of H-ZSM-5 zeolites with different concentrations of acid sites and silicalite did not change the curve profile. The mass loss started at 180 °C and went to approximately 400 °C. Marchal et al. observed similar behavior when they added intumescent formulations in ethylene vinyl acetate copolymer (EVA) [50]. The authors attributed the loss of mass to the reaction between APP and PER, producing a mixture of phosphate esters which decompose thermally, leading to the formation of *char* that simultaneously undergoes a swelling process. From 400 °C to 550 °C there was a reduction in the rate of mass loss, i.e., a gain in thermal stability due to the presence of *char*. Finally, starting at 550 °C, the decomposition of this intumescent layer and consequently of the composite could be detected.

The composites presented different percentages of residue at 750 °C. The PP+APP/PER composite presented 6.5% of residue. For composites containing zeo1, zeo2, zeo3 and sil, the percentages were 13.0%, 10.8%, 13.3% and 8.8%, respectively. The addition of H-ZSM-5 zeolites increased the amount of final residue compared to the PP + APP/PER composite, indicating an increase in the thermal stability of the intumescent layer. It appears that the concentration of acid sites and their accessibility had little influence on the thermal stability of the *char*. These characteristics of zeolites should be really important in the early stages of the formation of *char*, influencing the catalysis of the production of phosphate esters, precursors of the *char*. Regarding the percentage of residue obtained at high temperatures, studies with zeolites showed that their addition in polymeric matrices containing APP/PER led to a stabilization of the protective layer, which is attributed to the formation of aluminosilicophosphoric species, which would reduce the cleavage of P–O–C bonds present in the *char* [9,11,12,22,23,24,25,32]. These aluminosilicophosphoric species could also have been formed by the addition of H-ZSM-5 zeolites, regardless of the concentration and accessibility of acid sites. As previously reported, it is suggested that phosphoric acid production, resulting from the thermal decomposition of ammonium polyphosphate, leads to the destruction of the zeolitic structure [22]. This corroborates the hypothesis that the concentration of acid sites and their accessibility in H-ZSM-5 zeolites has little influence on thermal stability, and that the significant increase in the thermal stability of the protective layer is related to the formation of aluminosilicophosphoric species. Regarding the composite containing silicalite, the residue was slightly higher than that obtained from the composite PP + APP/PER and smaller than those observed for the composites containing H-ZSM-5 zeolites. It can be assumed that the absence of aluminum in its structure may have decreased the thermal stability of the protective layer, because the aluminosilicophosphoric species could be not formed. The increase of the residue at 750 °C from 6.5% to 8.8% with the addition of silicalite in the PP+APP/PER composite should simply be related to the amount of silicalite, which has high thermal stability, added to the composite at a concentration of 3%. 

#### 3.2.5. Heating Microscopy Analysis

**Figure 5:**
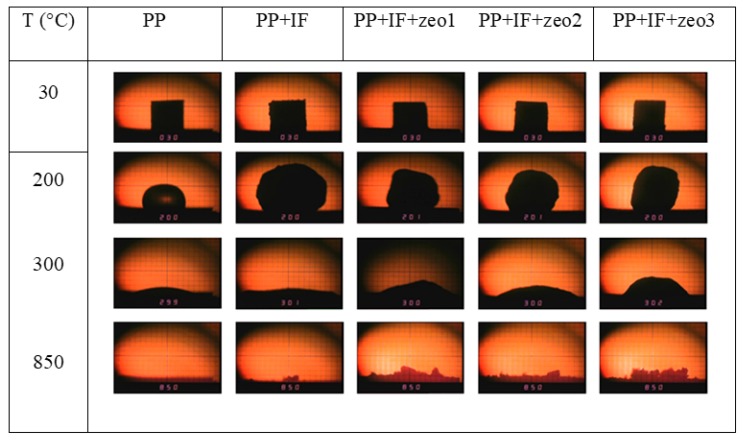
Images of the formation of the intumescent layer by heating microscopy technique.

Heating microscopy allows visualization of the intumescent layer formation in situ. The images of the composites containing the zeolites at 30, 200, 300 and 850 °C are shown in Figure 5. The test started at 30 °C, where it is possible to observe the cubic shape of the specimens. At 200 °C, the melting of the PP polymer was noted, with the center of the specimen becoming transparent due to the loss of the crystalline phase of the polymer chain. In the presence of the intumescent formulation, with or without the addition of zeolites, it was observed the beginning of the formation of the protective layer and its swelling. At 300 °C, PP and PP + APP/PER showed higher thermal degradation compared to composites containing zeolites. At 850 °C, PP was completely degraded and the PP + APP/PER showed a very small and poorly structured residue, different from the composites containing zeolite, which exhibited a higher thermal stability. This result corroborates the data obtained by TGA (Figure 4), which showed that the addition of zeolites, regardless of the concentration and accessibility of acid sites, increased the stability of the protective layer. At 750 °C, the percentage of residue obtained from composites containing zeo1 and zeo3, measured by TGA, was slightly higher than the percentage obtained from PP + APP/PER + zeo2. In Figure 5 it can be seen that the volume of the PP + APP/PER + zeo2 specimen was slightly smaller than the composites containing zeo1 and zeo3.

## 4. Conclusions

This work analyzed the effect of the concentration and accessibility of acid sites of H-ZSM-5 zeolites on synergistic action with an intumescent formulation composed of ammonium polyphosphate and pentaerythritol. The characterization of the zeolites obtained after desilication treatments showed that a large volume of mesopores was generated without a significant reduction in micropore volume and surface area. The extension of the time of the desilication treatment did not alter the volume of mesopores, but increased the amount of acid sites. The treatment for 30 min resulted in a concentration 3 times higher than that of the starting material, while after 2 h the increase was 6 times higher.

Limiting oxygen index and glow-wire tests showed that the addition of H-ZSM-5 zeolites improved the flame-retardant properties of the composites containing ammonium polyphosphate and pentaerythritol. In addition, the increment in the concentration and accessibility of the acid sites of H-ZSM-5 zeolites promoted an increase in the synergistic action with the intumescent formulation, which may result from the catalysis of the reaction between ammonium polyphosphate and pentaerythritol producing the precursors of the intumescent layer. On the other hand, heating microscopy and thermogravimetric analysis showed that the addition of H-ZSM-5 zeolites increased the thermal stability of the protective layer, but the concentration and accessibility of the acid sites seemed to have little effect on the amount of produced residue. In this case, the formation of aluminosilicophosphoric species should have a greater effect on the thermal stability of the protective layer.

## Figures and Tables

**Table 1:** Results of the textural characterization of H-ZSM-5 zeolites and silicalite.

Sample	Surface Area ^1^ (m^2^ g^−1^)	Micropore Volume ^2^ (cm^3^ g^−1^)	Mesopore Volume ^2^ (cm^3^ g^−1^)	Mesopore/ Micropore	Average Pore Diameter ^3^ (nm)
zeo1	425	0.12	0.14	1.17	6.40
zeo2	458	0.10	0.46	4.60	11.10
zeo3	369	0.11	0.40	5.00	11.46
Sil	294	0.12	0.10	0.83	2.41

^1^ BET surface area; ^2^ calculated by t-plot method; ^3^ BJH desorption average pore diameter (4V/A).

**Table 2:** Results of UL-94 tests for the samples of polypropylene composites.

Sample	UL-94	
	without APP/PER	with APP/PER
PP	NC ^1^	V0
PP + zeo1	NC ^1^	V0
PP + zeo2	NC ^1^	V0
PP + zeo3	NC ^1^	V0
PP + sil	NC ^1^	V0

^1^ The specimen was burned out and thus not classified.

**Table 3:** Results of the LOI tests for the polypropylene composites.

Sample	Mesopore/Micropore	Acidity (μmol NH_3_ g^−1^)	LOI % (±1) ^1^	
			without APP/PER	with APP/PER
PP	-	-	17	31
PP + zeo1	1.17	502	17	33
PP + zeo2	4.60	1674	17	32
PP + zeo3	5.00	3131	17	35
PP + sil	0.83	not detected	17	29

^1^ accuracy is ±1%.

**Table 4:** Values of GWFI and GWIT for the polypropylene composites.

Sample	GWFI (°C)	GWIT (°C)
PP	650	700
PP + IF	850	800
PP + IF + zeo1	960	850
PP + IF + zeo2	960	875
PP + IF + zeo3	960	875
PP + IF + sil	960	850

PP: polypropylene; IF: intumescent formulation.
